# Predicting Peptide Binding Affinities to MHC Molecules Using a Modified Semi-Empirical Scoring Function

**DOI:** 10.1371/journal.pone.0025055

**Published:** 2011-09-22

**Authors:** Webber W. P. Liao, Jonathan W. Arthur

**Affiliations:** 1 Sydney Medical School, University of Sydney, Sydney, New South Wales, Australia; 2 Children's Medical Research Institute, Sydney, New South Wales, Australia; Russian Academy of Sciences, Institute for Biological Instrumentation, Russian Federation

## Abstract

The Major Histocompatibility Complex (MHC) plays an important role in the human immune system. The MHC is involved in the antigen presentation system assisting T cells to identify foreign or pathogenic proteins. However, an MHC molecule binding a self-peptide may incorrectly trigger an immune response and cause an autoimmune disease, such as multiple sclerosis. Understanding the molecular mechanism of this process will greatly assist in determining the aetiology of various diseases and in the design of effective drugs. In the present study, we have used the Fresno semi-empirical scoring function and modify the approach to the prediction of peptide-MHC binding by using open-source and public domain software. We apply the method to HLA class II alleles DR15, DR1, and DR4, and the HLA class I allele HLA A2. Our analysis shows that using a large set of binding data and multiple crystal structures improves the predictive capability of the method. The performance of the method is also shown to be correlated to the structural similarity of the crystal structures used. We have exposed some of the obstacles faced by structure-based prediction methods and proposed possible solutions to those obstacles. It is envisaged that these obstacles need to be addressed before the performance of structure-based methods can be on par with the sequence-based methods.

## Introduction

Multiple sclerosis (MS) is a neurological disease characterised by inflammation and demyelination in the central nervous system. MS is regarded as an autoimmune disease by many researchers [1]–[5], however, the pathogenesis of the disease is not well understood. Genetic linkage analyses of MS patients have identified the DRB1*1501 and DQB1*0602 alleles of the Major Histocompatibility Complex (MHC) molecule as definite genetic risk factors [2], [5]. This has been confirmed in more recent genome wide association studies [6]. The MHC molecule is involved in the antigen presentation system and assists the T cells to identify pathogenic proteins. While the overall antigen presentation mechanism is reasonably well understood, the specificity and sensitivity of peptide binding to MHC molecules, and the binding of T-cells to the resultant complex, required to elicit an immune response, is not well defined. Deeper knowledge of the peptide binding process may help to isolate the cause of the disease and detect peptides with therapeutic potential.

Currently, there are three schools of MHC-peptide binding prediction methods based on the information and approach used in the prediction: sequence-motif (PSSM-) based, artificial intelligence- (AI-) based, and structure-based. The first two schools examine the patterns exhibited by the sequences of binding peptides, whereas structure-based methods study the relationship between the binding affinities and the structures of MHC-peptide complexes.

Early work on peptides that bind to MHC molecules observed patterns in the peptide sequences. Systemic analyses of the effects of amino acids on the peptide binding affinities provide the basis for position-specific scoring matrices to predict binding affinity [7]–[10]. More recently, many studies introduced artificial intelligence algorithms in the attempt to understand the subtle underlying patterns [11]–[14]. Due to the type of input, PSSM- and AI-based methods are sometimes generalised as sequence-based prediction methods [14].

In addition to sequence information, structure-based methods also incorporate additional structural information from experimental crystal structures of MHC-peptide complexes [15]–[21]. Usually the atomic coordinates of the MHC molecule are extracted from an experimental crystal structure as the frame template, and the atomic coordinates of the peptide from the same structure are used as the template for fitting new peptides. Once a structure fitted with a new peptide is constructed, the structure may be subjected to energy minimisation. Using the new structure, the distance between two atoms and the physiochemical properties of the atoms are used to determine if the interaction is beneficial or not to the binding.

Much effort has been put into developing sequence-based methods, which have shown considerable performance [8], [11], [14], [22]. On the other hand, the availability of experimentally determined structures allows structure-based methods to study the precise relationship between the structure and peptide binding specificity. The inclusion of structural information may reveal properties affecting the binding not obvious on the sequence level. Furthermore, the recent increase in the number of experimentally determined structures for MHC-peptide complexes is expected to provide further data to improve the performance of structure-based methods. A more detailed and comprehensive review of computational methods for predicting peptide binding to the MHC, particularly structure-based methods, has been written by Liao and Arthur [23].

Despite considerable research into the development of computational techniques for determining peptide binding to the MHC and successful predictions for some alleles, the performance of various binding prediction algorithms for MHC class II alleles, including DRB1*1501, is still relatively poor. Previously, Rognan *et al.* [24] had some success in predicting the binding affinity of peptides for the HLA A*0201 allele using a structure-based method. In the present study, we adopt the Fresno semi-empirical scoring function developed by Rognan *et al.* to study peptide binding to MHC class I and II alleles in order to improve the computational prediction of peptide binding to DRB1*1501.

## Results

### Validation of the prediction method

In this study, we adapted the semi-empirical method for predicting peptide binding affinity for MHC class I molecules originally proposed by Rognan *et al.* [24]. The public domain software packages MolProbity [25] and SCWRL 4 [26] were used instead of SYBYL BIOPOLYMER to add hydrogen atoms to the crystal structures and predict peptide side chain atomic positions. The modelling algorithm was implemented in PERL and R was used to perform the partial-least-square regression analysis with leave-one-out cross-validation.

The open source adaptation of the protocol was tested using the original five HLA-A0201 (A2) structures (the Madden structures) used by Rognan *et al.* Table 1 compares the experimental free energy of binding with the theoretical values of Rognan *et al.* and our analysis. In each case, our prediction more accurately estimates the experimental free energy of binding. The cross-validation correlation score, *q^2^*, was excellent at 0.971 and the standard error of prediction, *S_press_*, was appropriately low at 0.727. In comparison, Rognan *et al.* achieved a *q^2^* value of 0.895 and a *S_press_* value of 3.448. Thus, we established that our approach, using open source equivalents and our own PERL implementation of the Fresno scoring function, performs better than the original implementation.

**Table 1:** Comparison of the free energies for five HLA-A*0201 structures.

Peptide	PDB ID	ΔG_bind_, kJ/mol		
		Experimental^a^	Rognan^b^	Predicted^c^
TLTSCNTSV	1HHG	−37.32	−36.85 (−0.47)	−37.19 (−0.13)
FLPSDFFPSV	1HHH	−48.45	−48.56 (+0.11)	−48.41 (−0.04)
GILGFVFTL	1HHI	−46.94	−47.03 (+0.09)	−47.01 (+0.07)
ILKEPVHGV	1HHJ	−37.60	−38.96 (+1.36)	−37.74 (+0.14)
LLFGYPVYV	1HHK	−45.48	−45.57 (−0.09)	−45.43 (−0.05)

a Experimental values from the original publications.

b Predictions made by Rognan *et al.* in the original Fresno implementation; the deviations from the experimental values are included in parentheses.

c Our predictions; the deviations from the experimental values are included in parentheses.

Validation of our open source adaption of the method is crucial to ensure the integrity of our PERL implementation of the technique and the alternate use of open source applications. By repeating the analysis of Rognan *et al.*, we were able to show that our open source adaptation of the method reproduces the results of the original analysis, thus validating our adaptation. In fact, our approach generates slightly more accurate predictions than the original method.

### Prediction of peptide binding to HLA-DR15

Having validated the prediction method, we applied the procedure to the prediction of the free energy of peptide binding in HLA-DRB1*1501 (DR15). The HLA-DR15 allele of the MHC is a major genetic risk factor for MS. Our aim here was to use the method developed and validated above to predict peptide binding in this allele as a step to understanding the role this allele plays in the pathogenesis of MS.

There are only two experimentally determined structures for HLA-DR15: 1YMM and 1BX2. 1YMM was chosen as a reference structure as it was the most recently published crystal structure. The AntiJen database contains 188 entries of peptides with peptide binding data for HLA-DR15. Of these, only twenty peptides were fourteen amino acids in length as required to match the length of the peptide in the 1YMM reference structure. These peptides are shown in Table 2.

**Table 2:** All twenty 14-mer peptides with experimental binding data in regard to HLA-DR15 extracted from AntiJen.

Peptide	IC_50_ (nmol)	Temp (°C)
ADTISSYFVGKMYF [40]	160	37
DENPVVHFFKNIVT [41]	4.6	37
DTISSYFVGKMYFN [41]	780	37
ENPVVHFFKNIVTA [41]	12	37
FNLIDTKCYKLEHP [41]	35000	37
GKMYFNLIDTKCYK [41]	33000	37
HFFKNIVTPRTPPY [41]	405	37
ISSYFVGKMYFNLI [41]	1600	37
KMYFNLIDTKCYKL [41]	68000	37
KNSADTISSYFVGK [41]	210	37
MYFNLIDTKCYKLE [41]	6500	37
NLIDTKCYKLEHPV [41]	40000	37
NPVVHFFKNIVTPR [41]	6.8	37
NSADTISSYFVGKM [41]	330	37
SADTISSYFVGKMY [41]	230	37
SSYFVGKMYFNLID [41]	1600	37
SYFVGKMYFNLIDT [41]	400	37
TISSYFVGKMYFNL [41]	190	37
YFNLIDTKCYKLEH [41]	15000	37
YFVGKMYFNLIDTK [41]	33000	37

Each peptide was modelled in the binding groove of the MHC molecule and the resulting structure used to determine the terms of Fresno scoring function (equation 2). The resulting equations for all twenty peptides were then subjected to the statistical analysis to determine the regression coefficients. These regression coefficients are then used to predict the theoretical binding free energy for each peptide for comparison with the experimental data. After the cross-validation analysis, the *q^2^* value for the analysis was 0.243 and *S_press_* was 6.429 confirming the prediction method was unable to accurately reproduce binding free energies for peptides in HLA-DR15.

To confirm this result was not due to an anomaly with the 1YMM structure, we also repeated the analysis with the 1BX2 structure. Similar results were obtained (data not shown).

Thus, the success of the scoring function in reproducing, and slightly improving, the results of Rognan *et al.* with the class I A*0201 allele, was not seen when working with the class II DRB1*1501 allele. This prompted us to a detailed examination of the Rognan *et al.* scoring function and its applications to assess the efficacy of the method in different circumstances.

### Effect of data quantity on prediction accuracy

One possible explanation for the failure to adequately predict binding free energies in HLA-DR15 compared to the success in predictions with HLA-A2 may relate to class II MHC molecules requiring a larger set of binding data to better predict peptide binding. However, as noted above, only twenty peptides of appropriate size are contained in the AntiJen database for HLA-DR2.

In order to test this hypothesis, we considered HLA-DRB1*0101 (DR1) and HLA-DRB1*0401 (DR4): the two most studied class II alleles. Multiple PDB entries can be found for both HLA-DR1 and HLA-DR4 alleles. The most recently published structures with the best resolution were used as reference structures (1FTY and 1J8H). Both these alleles have more peptides with experimental binding data in the AntiJen database than HLA-DR15 with 74 peptides from 11 studies meeting the selection criteria for HLA-DR1 and 58 usable peptides from the same study for HLA-DR4.

The calculated *q^2^* and *S_press_* values for HLA-DR1 were 0.275 and 7.795 respectively. The calculated *q^2^* and *S_press_* values for HLA-DR4 were 0.283 and 6.390. Thus, using larger peptide binding data reference sets results in a modest improvement in both the cross-validation correlation score and the standard error of prediction to DR-15. However, the former remains low, and the latter high, indicating that the predictive capacity of the method remains poor. This suggests that while the quantity of peptide binding data does have an impact on the predictive ability of the scoring function, it is not the primary factor.

### Effect of MHC class on prediction accuracy

Another possible factor affecting the prediction may be the class of MHC molecule used as the reference structure. The original method of Rognan *et al.* was developed and tested on MHC class I, and allele A*0201 in particular. It is possible that the more open topology of the MHC class II structure means the approach is not suitable, at least in its current form, for class II molecules. To explore this possibility, we attempted to duplicate our experiments above, but with class I molecules, and the A*0201 allele in particular.

As a reference structure, we chose 2GTW for the HLA-A2 allele [27]. This structure is not one of the five Madden structures, has a high resolution, and was published recently. A list of 174 peptides from 22 studies was extracted from the AntiJen database. Thus, our selection replicates the selection we made previously for a class II allele.

The calculated *q^2^* and *S_press_* values using the structure 2GTW were 0.01974 and 6.037. Thus, even using a class I structure, with a large set of peptide binding data, the technique does not achieve good predictive capability. To confirm this, we repeated the experiment using one of the five Madden structures as a reference. Since the peptide in the structure 1HHH is longer (decamer) than the other structures (nonamers), the 1HHH data was incorporated in two ways. The peptide of 1HHH was either truncated at the N-terminal or the C-terminal of the peptide in order to fit into the other structures with nonamers, or the peptide was excluded from the analysis completely. The MHC structure of 1HHH structure was not used at all, since peptides from the other structures will not fit. The procedure was repeated for each of the four structures (1HHG, 1HHI, 1HHJ, and 1HHK).

When the peptide from 1HHH was not used (*i.e.* only four peptides were used as input data), 1HHG, 1HHI, and 1HHJ returned low *q^2^* values suggesting no predictive capability for the technique. The *q^2^* and *S_press_* values for 1HHK, however, were significantly better at 0.7897 and 1.75, although still not nearly as good as the values seen in the validation study. When the peptide from 1HHH was used, none of the reference structures was able to return a good result.

The favourable result for 1HHK presented a possible reason for the performance of validation study. 1HHK was therefore used as the reference structure in a further analysis under the same conditions used for 2GTW. However, this analysis gave *q^2^* and *S_press_* values of 0.002 and 6.083.

### Predictive capability is dependent on quantity of structural data

The previous experiments consistently showed poor predictive capability for the approach, despite the remarkable success of the approach in the validation study. A final point of difference between the experiments is that the validation study uses five reference structures *i.e.*, in calculating the terms and thence the regression coefficients, the atomic distances used are those of the peptide in its native crystal structure. In contrast, the other studies use peptides modeled in a single reference crystal structure.

Since calculation of the free energy of binding is based on the reference structure, if the predicted structure is different from how the peptide binds the MHC molecule natively, it may damage the predictive performance of the method. Thus, using a large set of reference structures simultaneously may provide more structural information and thus lead to better predictions.

To test the hypothesis, we searched PDB for HLA-A2 structures with one of the 174 peptides previously collected from AntiJen database, and found 17 structures, including 1HHJ (Table 3). Fourteen of them share one of three common peptides (ILKEPVHGV, NLVPMVATV, and SLLMWITQC) with other structures. Thus, we used various combinations of 6 structures, consisting of the 3 unique structures and a combination of three structures chosen from the 14 structures sharing the three common peptides, such that only one structure with each peptide was used.

**Table 3:** List of PDB entries and corresponding peptide binding data.

Peptide	Temperature (°C)	IC_50_ (nmol)	PDB
AAGIGILTV [42]	4	0.00008	2GUO
FLWGPRALV [42]	4	0.0000021	1QEW
ILKEPVHGV [43]	4	0.000008	1AKJ
ILKEPVHGV	4	0.000008	1HHJ
ILKEPVHGV	4	0.000008	1P7Q
ILKEPVHGV	4	0.000008	2X4U
IMDQVPFSV [44]	26	0.00000654	1TVH
NLVPMVATV [45]	4	0.0000125	2X4R
NLVPMVATV	4	0.0000125	3GSN
NLVPMVATV	4	0.0000125	3GSO
SLLMWITQC [46]	37	0.00002107	1S9W
SLLMWITQC	37	0.00002107	2BNR
SLLMWITQC	37	0.00002107	2F53
SLLMWITQC	37	0.00002107	2F54
SLLMWITQC	37	0.00002107	2P5E
SLLMWITQC	37	0.00002107	2P5W
SLLMWITQC	37	0.00002107	2PYE

**Figure 1:**
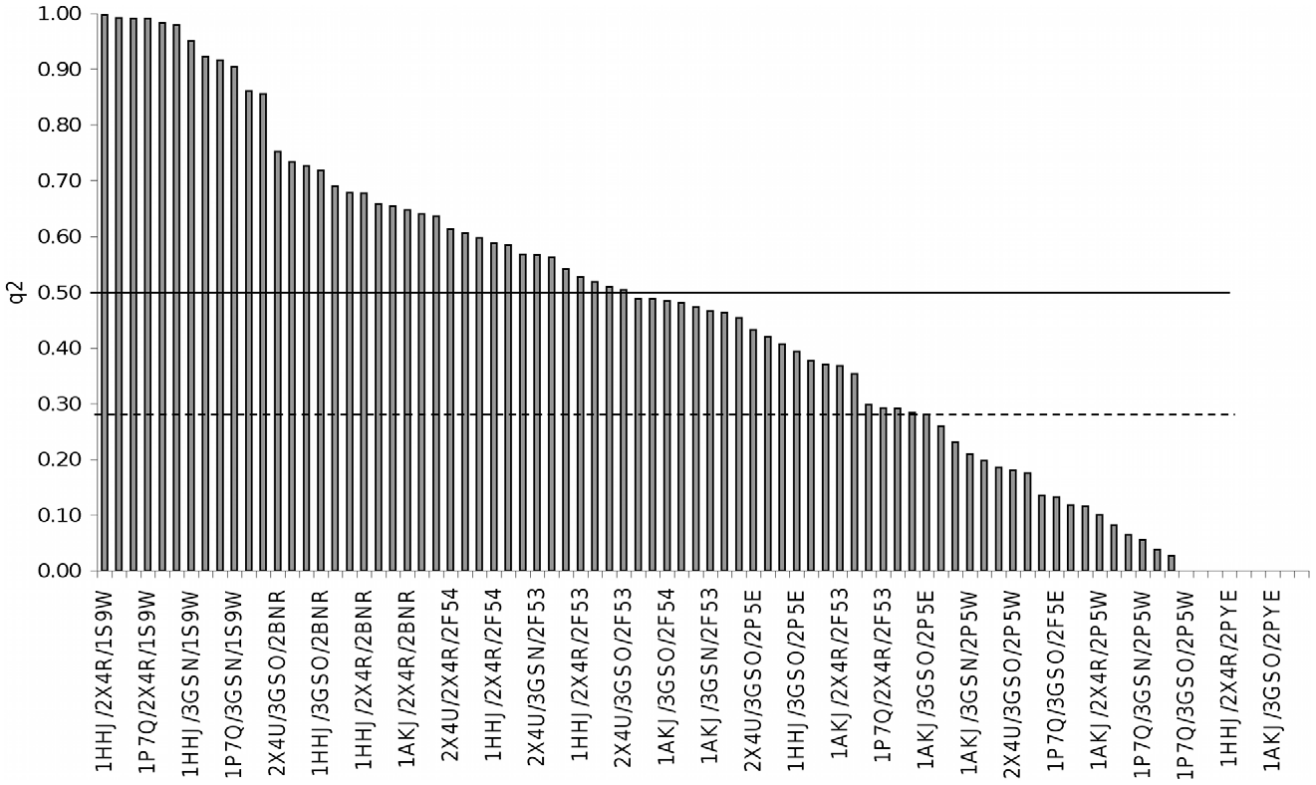
Spread of q2 values for different combinations of reference structures. 37 out of 84 combinations of reference structures (44%) achieved a q2 value greater than 0.5 and 57 (68%) achieved a q2 value greater than 0.283. which was the best predictive performance for analyses using only one reference structure.

The *q^2^* and *S_press_* values varied between 0.998 to complete randomness. However, most combinations (57 combinations) showed improvement over the best of the previous analyses using a single reference structure (*q^2^* value of 0.283) and nearly half of the combinations (37 combinations) achieved a *q^2^* value greater than 0.5 (Fig. 1). This supports our hypothesis that using multiple reference structures will boost the prediction performance.

Yet, the effect is not definitive. While most sets of reference structures generate better results than a single reference structure, the predictive capability still varies depending on the reference set chosen, with many reference sets still showing less than adequate predictive capability, despite improvements over single reference structure methods.

To examine the potential impact of different structural characteristics on the predictive performance, we explored the correlation between *q^2^* and *S_press_* values and various characteristics of the structures (Table 4). The first of these was the average root mean square deviation (RMSD) of the reference structures. The RMSD was calculated for all the combinations used in the analysis using the atoms from the MHC molecule alone, the peptide alone, and the whole structure. The RMSD scores were calculated for all pairs of structures in the set of reference structures and the results averaged to give a mean RMSD score for the set The RMSD scores were compared to the corresponding *q^2^* and *S_press_* values using Spearman's rank correlation. Secondly, the *q^2^* and *S_press_* values were also compared to the average resolution of the structures using Spearman's rank correlation. A correlation coefficient of 1 (or −1) indicates perfect correlation in the same (or opposite) direction. A value of 0 indicates no correlation.

**Table 4:** Comparison between *q^2^* and *S_press_* to the RMSD score and the resolution of structures.

	*q^2^*	*S_press_*
RMSD	−0.607	0.604
RMSD_MHC_	−0.579	0.577
RMSD_peptide_	0.076	−0.080
Average resolution	−0.103	0.105

Three RMSD scores were calculated based on the use of the structures. RMSD_MHC_ is the RMSD for the structure of the MHC molecule alone, RMSD_peptide_ is the RMSD for the structure of peptide alone, and the RMSD for the whole structure.

**Figure 2:**
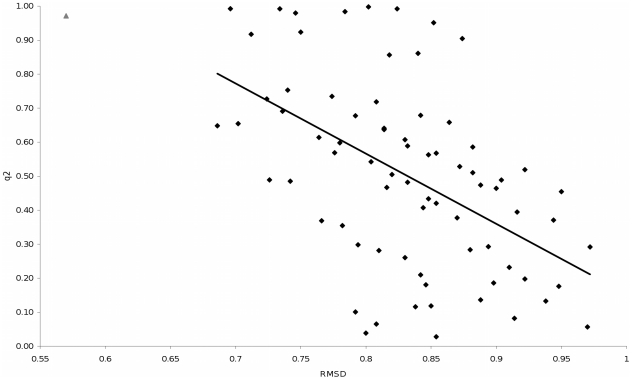
The comparison of q2 values and RMSD scores shows a general negative correlation. The point for the Madden structures is the grey triangle located towards the top left of the figure.

The Spearman's coefficient between the *q^2^* values and the RMSD scores shows an intermediate correlation between average RMSD score and *q^2^* value with a small average RMSD between the structures giving rise to a high q^2^ value, and thus better predictive performance for the approach (Fig. 2). This is also the case for the five Madden structures used in the original Fresno study. The average RMSD score of the five Madden structures was 0.57, which is better than all of the combinations used in the analysis, giving rise to the high *q^2^* value and predictive performance in the original study. The correlation between the *q^2^* values and the RMSD_MHC_ scores suggests that the correlation is primarily attributed to the structure of the MHC molecule.

On the other hand, little correlation was seen between the average resolution of the structures and the *q^2^* values. This suggests that depth of resolution of the reference structures is not critical to the predictive performance of the method.

## Discussion

In this series of experiments, we have shown that our implementation of the Fresno scoring function, using open source/free software, reproduces the results of Rognan *et al.* and, in fact, performs slightly better than the original implementation. However, when the number of reference structures used is reduced to one, the performance of the scoring function is greatly diminished, even if a large set of peptide binding data is used. This indicates that either MHC molecules assume quite different positions whilst binding to different peptides or that the theoretical approach used to predict peptide binding is quite sensitive to small changes in MHC structure. If a MHC molecule binds to the peptides more or less in the same way, the differences between structures should be minimal, and the scoring function should still be able to predict the binding affinity albeit with a less satisfying performance. On the other hand, if the MHC molecule assume different positions when binding to different peptides, multiple structures will be required to effectively sample all possible confirmations used as a basis for the semi-empirical model. Our experiments demonstrate this to be the case. When only one of the five structures used in the original Fresno study was used to analyse the binding affinities of all the peptides, only one structure could be used to achieve a good performance. Nonetheless, this performance was still worse than using all five reference structures. We also showed that even when one of the best structures for HLA-A2 is used as the reference structure, the prediction performance was still less than ideal, but when more reference structures were employed the *q^2^* value can reach over 0.9. It is therefore important to consider various binding confirmations when constructing a free energy scoring function.

The best solution is to determine the structure for all binding peptides used in both establishing the regression coefficients for the scoring function as well as those whose binding free energy is to be predicted. However, this need for structural information for each peptide being considered makes it effectively impossible to use the method in large scale computational studies, such as an exhaustive scan of all possible peptides to predict potential epitopes for the MHC molecule.

Two further approaches offer a potential solution to this problem. The first is to obtain a large set of structures and use the structure with the most similar peptide for the peptides that do not have an experimentally determined structure. The other approach is to derive a “consensus structure” by averaging all the available structures. A consensus structure may sacrifice accuracy for some peptides but will hopefully be able to fit most peptides within a tolerable error level. Due to the nature of these approaches, the first may provide higher accuracy, however, the second approach should be easier to implement.

Another obstacle for structure-based methods is the reduced set of binding data. While sequence-based methods can simply categorise peptides into binders or non-binders based on the IC_50_ values, structure-based methods often rely on precise input, which excludes implicit values, such as strong, intermediate, and weak binding. Moreover, there is discrepancy in the binding data for many peptides due to various experimental settings. Any slight change in the input can produce a different result, and a large inconsistency in the input can render the result useless. However, discrepancies may be introduced in two areas: the detection method and the choice of competing peptide in the competitive assay. There are two detection methods based on the labelling tag, either fluorescence or radioactive isotopes, used to label the target peptide. While the two methods share the same principle, the readings can vary greatly and a difference is observed between two studies using different labelling method. In addition to the detection methods, the choice of competing peptide is also an important factor in determining the IC_50_ value. When two competing assays are performed using the same detection method and same experimental conditions but different competing peptides, the relative binding affinity of the two competing peptides will affect the resulting binding affinity of the target peptide. If the first competing peptide is a better binder than the second competing peptide, there will be a difference in the resulting IC_50_ values. This may be the reason why two studies may arrive at different IC_50_ values even though all the other experimental conditions appear to be the same.

It is possible to include the implicit values if the scoring function is classification-based, where input is classified into weak, intermediate, or strong binders. Although this will inevitably reduce the information used to deduce the scoring function and reduce the accuracy of the scoring function, using a classification-based approach will allow more input data. This may compensate for the loss of specific binding information. Unfortunately, it is impossible to resolve the discrepancy introduced by using different competing peptides; prior knowledge will be required to be able to choose one IC_50_ value over another.

In conclusion, the present study implemented the Fresno scoring function using open source and free software. We have also looked at some of the obstacles faced by researchers in the attempt to develop free energy scoring functions. Currently, sequence-based methods exploring binding motif or utilising artificial intelligence are leading the race to accurately predict peptide binding affinity. However, sequence-based methods do not face the same obstacles as structure-based methods, as they do not utilise structural information and tend to be classification based. While structure-based methods are not so far behind, it is foreseeable that these obstacles need to be addressed before the performance of structure-based methods can be on par with the sequence-based methods.

## Materials and Methods

### Preparation of MHC structures

A list of experimentally determined structures of the MHC-peptide complex for alleles HLA-A*0201, HLA-DRB1*0101, HLA-DRB1*0401, and HLA-DRB1*1501 (Table 5) were collected from the Protein Data Bank [28]. For analyses where only one structure was used the most recent structure with best resolution was used. Structures, referred to as the Madden structures hereafter, used by Rognan *et al.* in their study (1HHG, 1HHH, 1HHI, 1HHJ, 1HHK) were also obtained from the PDB [29].

**Table 5:** Experimental crystal structures used in the present study.

Allele	PDB ID
HLA-A*0201 (Madden structures)^*^	1HHG, 1HHH, 1HHI, 1HHJ,1HHK [29]
HLA-A*0201	1AKJ, 1B0R, 1OGA, 1P7Q, 1QEW, 1S9W, 1TVH, 2BNR, 2BNQ, 2F53, 2F54, 2GTW, 2GT9, 2GUO, 2P5E, 2P5W, 2PYE, 2X4U, 2X4R, 3GSN, 3GSO [27], [47]–[57]
HLA-DRB1*0101	1FYT [58]
HLA-DRB1*0401	1J8H [59]
HLA-DRB1*1501	1YMM, 1BX2 [60]–[61]

The Madden structures were the five structures used in the original Fresno study.

Each crystal structure gives the positional information of the atoms of the MHC molecule and a peptide of particular sequence bound to the MHC molecule. In order to study the binding affinity of other peptides, the structure of a new peptide, bound to the same MHC molecule, is determined from the existing structure by using the same positions for the backbone atoms and rebuilding the side chains in the context of the MHC molecule. In the present study, the side chain rebuilding was performed using SCWRL 4 [26]. SCWRL 4 preserves the positions of the backbone atoms for the new peptide. It then attempts to predict the positions of the side-chain atoms for the new peptide while considering steric effects of the surrounding framework: in this case, the MHC molecule. Once a structure with the new peptide was constructed, hydrogen atoms were added using MolProbity 3.14 [30].

### Preparation of peptide binding data

When the concentration of the binding peptide is sufficiently low, the dissociation constant can be represented by the inhibitory concentration (IC_50_): the concentration of inhibitor required to halve the level of binding of the substrate to the enzyme in a competitive assay. The free energy of binding can be calculated from the experimental temperature in Kelvin (T), the IC_50_ value, and the gas constant (R) according to equation 1.
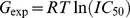
(1)


A list of peptides with known binding affinity was extracted from the AntiJen database for each allele [31]–[32]. The AntiJen database contains experimental binding data for peptides known to bind to MHC molecules. Only peptides with the same length as the peptide in the reference crystal structure were used; typically, these were nine amino acids long. Inconsistencies or implicit values in the data set, such as multiple IC_50_ values for individual peptides due to different experimental settings, were resolved by manual reference to the original citations. If there is inexplicable discrepancy, the peptides in question were excluded from the analysis. The experimental data for the five structures used in Rognan *et al.* were taken from their original publication [33].

### Calculation of the Scoring Function Terms

The Fresno free energy scoring function was previously described by Rognan *et al.* [24]. Briefly, there are five terms used by the Fresno scoring function (equation 2). Each term attempts to model the contribution to the binding energy made by a different atomic interaction.
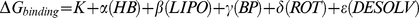
(2)


The first three terms describe the energies associated with hydrogen bonds (HB), the interactions between lipophilic atoms in the MHC molecule and the peptide (LIPO), and the unfavourable interactions between polar and lipophilic atoms (BP). The rotational term (ROT) estimates the loss of energy due to the freezing of the rotatable bonds of the peptide upon binding. Lastly, the desolvation term (DESOLV) considers the energies required to solvate the MHC molecule, the peptide, and the MHC-peptide complex. The equations and related details for calculating each term are given in Rognan *et al* [24] and Eldrige *et al.* [34].

### Calculation of the Regression Coefficients

The HB, LIPO, ROT, and BP terms were calculated using an adaptation of the Fresno scoring function developed in PERL. If the reference PDB file contained a bound T-cell receptor, this part of the file was removed prior to the analysis. The DESOLV term for all peptides was estimated using the DelPhi program [35]–[36]. The parameters were similar to those used by Rognan *et al.* The only difference being the atomic radii and the charges. Atomic radii and charges used in this study were taken from PARSE [37].

The values of all terms and the experimental free energy are used in a partial least square analysis using *R* and the *pls* package [38]–[39]. Regression coefficients were derived for each term and optimised for each dataset. The theoretical free energy was predicted using these regression coefficients. The cross-validation correlation score (*q^2^*) and standard error of prediction (*S_press_*) were calculated from leave-one-out cross-validation using the built-in functions (*R2* and *RMSEP*) from the *pls* package. *q^2^* estimates the accuracy of the model and *S_press_* estimates the error rate of the prediction. Thus a good prediction model should have *q^2^* close to 1 and a low *S_press_* value.

### Correlation Studies

The difference between protein structures was calculated in terms of root mean square deviation (RMSD) scores. Structures were superimposed and the RMSD values were calculated using the Discovery Studio Visualizer 3.0 by Accelrys. Correlation between the *q^2^* values and the RMSD scores were analysed using Spearman's rank correlation in R. Correlation between the *q^2^* values and the average resolution of structures, obtained from the PDB structure files, was calculated similarly.
